# Retraction Note: Thermal cooling efficacy of a solar water pump using Oldroyd-B (aluminum alloy-titanium alloy/engine oil) hybrid nanofluid by applying new version for the model of Buongiorno

**DOI:** 10.1038/s41598-025-85426-z

**Published:** 2025-01-14

**Authors:** Faisal Shahzad, Wasim Jamshed, Mohamed R. Eid, Rabia Safdar, Siti Suzilliana Putri Mohamed Isa, Sayed M. El Din, Nor Ain Azeany Mohd Nasir, Amjad Iqbal

**Affiliations:** 1 Department of Mathematics, Capital University of Science and Technology (CUST), Islamabad, 44000 Pakistan; 2 Department of Mathematics, Faculty of Science, New Valley University, Al-Kharga, 72511 Al-Wadi Al-Gadid Egypt; 3 Department of Mathematics, Faculty of Science, Northern Border University, Arar, 1321 Saudi Arabia; 4 Department of Mathematics, Lahore College for Women University, Lahore, 54000 Pakistan; 5 Institute for Mathematical Research, Universiti Putra Malaysia, UPM, 43400 Serdang, Seri Kembangan, Selangor Darul Ehsan Malaysia; 6 Center of Research, Faculty of Engineering, Future University in Egypt, New Cairo, 11835 Egypt; 7 Department of Mathematics, Centre for Defence Foundation Studies, Universiti Pertahanan Nasional Malaysia, Kem Sungai Besi, 57000 Kuala Lumpur, Malaysia; 8 Department of Materials Technologies, Faculty of Materials Engineering, Silesian University of Technology, 44-100 Gliwice, Poland; 9 CEMMPRE—Centre for Mechanical Engineering Materials and Processes, Department of Mechanical Engineering, University of Coimbra, Rua Luı’s Reis Santos, 3030-788 Coimbra, Portugal

Retraction of: *Scientific Reports* 10.1038/s41598-022-24294-3, published online 17 November 2022

The Editors have retracted this Article as it contains several nonstandard phrases for which the authors have not been able to provide a suitable explanation. In addition the Article shows evidence of authorship and citation irregularities, which the Authors were not able to satisfactorily explain. The Editors therefore no longer have confidence in the reliability of this Article and its metadata.

Authors Mohamed R. Eid, Siti Suzilliana Putru Mohamed Isa and Amjad Iqbal disagree with this retraction. The other Authors have not responded to correspondence regarding this retraction.

